# Mind-Body Therapies for Cancer Patients Living with Depression, Anxiety or Insomnia (MIRACLE): A Systematic Review with Individual Participant Data Network Meta-Analysis

**DOI:** 10.3390/mps4040076

**Published:** 2021-10-19

**Authors:** Yoann Birling, Sarah Nevitt, Deep Jyoti Bhuyan, Mingxian Jia, Fan Feng, Linda Ellen Carlson, Tiffany Pham, Jing Liu, Zahra Ayati, Liyi Nyiam, Zhichao Yu, Paul Fahey

**Affiliations:** 1 NICM Health Research Institute, Western Sydney University, Penrith 2751, Australia; Z.Ayati@westernsydney.edu.au; 2 Institute of Population Health, University of Liverpool, Liverpool L69 3GF, UK; Sarah.Nevitt@liverpool.ac.uk; 3 Institute of Chinese Medicine, China Resources Sanjiu Medical and Pharmaceutical Co., Ltd., Shenzhen 518110, China; mingxian.jia@hotmail.com; 4 Benson Henry Institute for Mind Body Medicine, Massachusetts General Hospital, Boston, MA 02114, USA; FFENG1@mgh.harvard.edu; 5 Department of Psychology, University of Calgary, Calgary, AB T2N 1N4, Canada; l.carlson@ucalgary.ca; 6 School of Health Sciences, Western Sydney University, Penrith 2751, Australia; 17119245@student.westernsydney.edu.au (T.P.); 19640192@student.westernsydney.edu.au (J.L.); zhichao.yu@westernsydney.edu.au (Z.Y.); p.fahey@westernsydney.edu.au (P.F.); 7 Pre-Disease Treatment Department, The Gulou Hospital of Traditional Chinese Medicine of Beijing, Beijing 100009, China; wanzhi_hino@yahoo.com; 8 The First Clinical Medical College, Nanjing University of Chinese Medicine, Nanjing 210023, China

**Keywords:** cancer, depression, anxiety, insomnia, yoga, tai chi, qigong, relaxation, mind-body, mindfulness

## Abstract

Depression, anxiety, and insomnia are common in cancer patients. Mind-body therapies (MBTs) are promising forms of treatment for cancer patients living with depression, anxiety, and insomnia. The objective of this study is to assess the effectiveness and acceptability of MBTs in cancer patients living with depression, anxiety, or insomnia. EMBase, PubMed, Cinahl, PsychINFO, IndMED, CSI-NISCAIR, CNKI, Clinicaltrial.gov, ChiCTR, and CTRI will be searched until October 2020 for relevant studies. Randomized controlled studies in which MBTs were tested in a cancer population will be selected. The authors of the selected studies will be contacted to obtain individual participant data. The participants who reached a defined clinical threshold for depression, anxiety, or insomnia will be selected for the three sub-studies on depression, anxiety, and insomnia, respectively. Pairwise and network meta-analyses will be used to assess the changes in depression, anxiety, sleep quality, and completion rate. We will assess the effect of the treatment dose (number and frequency of interventions) on effectiveness. The results of this study will inform clinical decision-making for the treatment of psychological disturbances in cancer patients. If MBTs are found effective, they will potentially be recommended as treatments for cancer patients with psychological symptoms.

## 1. Background

Psychological disturbances are highly prevalent in cancer patients. It is estimated that approximately 25%, 10%, and 28% of cancer patients suffer from clinical depression, anxiety, and insomnia, respectively [1,2], which is roughly double the rates of the general population [3,4,5,6]. This higher prevalence can be attributed to the shock caused by a cancer diagnosis [7], the burden of cancer treatment [8], and the correlations between different psychological symptoms and between these symptoms and fatigue and pain [9,10,11,12,13,14]. In this context, treatments targeting different aspects of physical and psychological health have a decisive advantage in the treatment of psychological disturbances in cancer patients.

Depression, anxiety, and insomnia significantly impair the quality of life of cancer patients. Psychological health strongly predicts quality of life in cancer patients [15] and impacts treatment outcomes [16,17,18]. Moreover, the hyperactivity of the hypothalamus-pituitary axis (HPA) present in psychological disturbances weakens the immune system, affecting its ability to fight cancer [19]. For the above reasons, psychological disturbances increase mortality in cancer patients [20,21]. The effective management of psychological symptoms improves both adherence to anti-cancer treatment and mortality rates [22,23,24].

Psychological disturbances in cancer patients are mostly treated with pharmacological drugs, such as hypnotics, antidepressants, and anxiolytics [25,26]. Although the efficacy of these treatments has been proven, they tend to induce adverse reactions, such as morning sedation, anterograde amnesia, falls, undesired sleep behavior, gastrointestinal symptoms, weight gain, sexual dysfunction, and anxiety [27,28,29,30,31,32]. Moreover, long-term use of psychotropic medications increases the risk of dementia, diabetes, and cancer, and increases the overall mortality rates [33,34,35,36,37,38,39]. Patients who suffer from psychological disturbances prefer non-pharmacological approaches [40,41].

Mind-body therapies (MBTs) are practices that aim to improve health through mindfulness, breathing exercises, postures, movements, and relaxation [42,43]. They include ancient practices, such as yoga, qi gong, and tai chi, and modern therapies, such as mindfulness-based stress reduction (MBSR) training and progressive muscular relaxation (PMR). Due to their broad effect on well-being, including improved mood, reduced pain, higher energy levels, and higher social functioning, MBTs seem to be ideal treatment approaches for psychological disturbances in cancer patients [44,45,46,47,48,49,50]. They are also relatively safe and widely accessible to cancer patients in many parts of the world.

Previous systematic reviews have shown that MBTs can improve the moods and sleep quality levels of cancer patients [51,52,53,54,55,56]. However, these reviews included both participants living with and not living with psychological disturbances. They did not report the acceptability of the interventions, which is an important aspect to consider in decision-making, as cancer patients have limited time and energy. They did not report either the treatment dose (number and frequency of treatments) necessary to achieve clinically significant improvements. Thus, a review of the effectiveness and acceptability of MBTs in cancer patients living with depression, anxiety, or insomnia is needed.

Most randomized-controlled trials testing MBTs for cancer patients have not specifically selected cancer patients living with psychological disturbances [57]. Therefore, it is crucial to collect the individual participant data (IPD) from these RCTs and conduct a meta-analysis with the data from participants who meet the clinical thresholds for depression, anxiety, and/or insomnia. Different MBTs are rarely compared to each other in clinical trials; therefore, a network meta-analysis (NMA) is necessary to assess the relative effectiveness of different MBTs.

## 2. Methods

**Figure 1:**
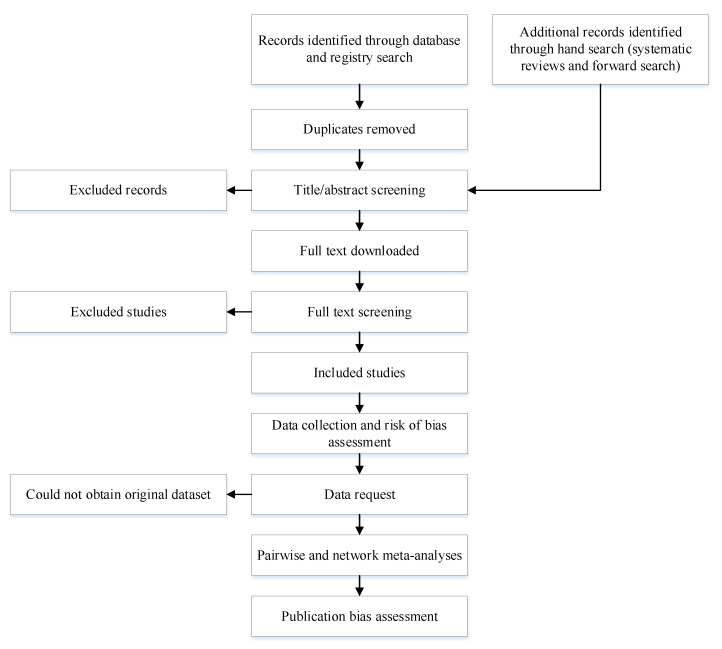
Methodological processes of the systematic review.

This study will be conducted according to the Cochrane guidelines on IPD systematic reviews and NMA systematic reviews [58,59]. The protocol was registered in PROSPERO (CRD42021240595). The results of this review will be published separately for each of the sub-studies. The Human Research Ethics Committee of Western Sydney University, Australia exempted the present review from ethical review. The methodological processes of the study are reported in Figure 1.

### 2.1. Study Selection

#### 2.1.1. Study Design

Studies in which participants were randomly allocated will be included. Both parallel and cross-over studies will be included. For cross-over studies, only the first period of the study will be included.

#### 2.1.2. Participants

We will include adults diagnosed with cancer—regardless of the type of cancer, cancer stage, type of treatment received, or stage of treatment. However, studies conducted specifically around a medical procedure (e.g., biopsy, surgery) will be excluded.

For the sub-study on depression, we will select the participants who meet the clinical threshold for depression on a validated tool. The accepted assessment tools with validated cut-off scores are: (1) PHQ-9 score ≥ 10 [60]; (2) CES-D score ≥ 20 [61]; (3) BDI score ≥ 16 [62]; (4) HAMD score ≥ 12 [63]; (5) POMS depression score ≥ 7 [64]; (6) PHQ-2 score ≥ 2 [60]; (7) HADS-D ≥ 11 [65]; (8) PROMIS depression scale score ≥ 8 [66]; (9) DASS-D score ≥ 10 [67]; (10) SCL-90 depression score ≥ 25 [63]; (11) MADRS score ≥ 12 [68]; and (12) BDI-II score ≥ 14 [69]. If the participant was assessed with two of these tools, the decision will be based on one assessment tool, in the above order of priority.

For the sub-study on anxiety, we will select the participants who meet the clinical threshold for anxiety on a validated tool. The accepted assessment tools with validated cut-off scores are: (1) GAD-7 score ≥ 8 [70]; (2) HAMA score ≥ 11 [71]; (3) BAI score ≥ 12 [72]; (4) HADS-A ≥ 11 [65]; (5) STAI-S ≥ 51 [73]; STAI-T ≥ 53 [73]; (6) DASS-A ≥ 8 [67]; (7) PROMIS anxiety score ≥ 8 [66]; (8) GAD-2 ≥ 3 [70]; (9) POMS anxiety score ≥ 16 [74]; and (10) PSWQ ≥ 62 [75]. If the participant was assessed with two of these tools, the decision will be based on one assessment tool, in the above order of priority.

For the sub-study on insomnia, we will select the participants who meet the clinical threshold for insomnia on a validated tool. The acceptable assessment tools with validated cut-off scores are: (1) ISI score ≥ 11 [76]; (2) PSQI score ≥ 6 [77]; and (3) AISI score ≥ 6 [78]. If the participant was assessed with two of these tools, the decision will be based on one assessment tool, in the above order of priority.

#### 2.1.3. Interventions

MBTs are defined as therapies that emphasize the use of the brain in conjunction with the body to assist the healing process [79]. Meditation, mindfulness-based therapies, mindfulness-based cognitive therapy, yoga-based interventions, tai chi, qigong, visualization and imagery, hypnosis and self-suggestion, art therapy, music therapy, dance therapy, biofeedback, and relaxation training—including compound interventions of the above—will be included in this review. Psychotherapy, exercise therapy, and acupuncture will not be accepted. Compound interventions, including MBT and counseling, education, and/or diet, will not be accepted except in cases where the non-MBT interventions are minor. In the NMA, the interventions will be grouped by label (e.g., “yoga”, “qigong”) rather than treatment components (e.g., breathing techniques, mindfulness). For example, two interventions respectively labeled “yoga” and “qigong” by the study authors but involving the same components (e.g., breathing techniques and posture) will be grouped under two different categories.

#### 2.1.4. Controls

Inactive controls and active controls, such as wait-list, no-treatment, usual care, exercise therapy, psychoeducation, sham MBT, or another MBT, will be considered as appropriate control interventions.

#### 2.1.5. Outcomes

For each sub-study, the primary outcomes will be, respectively, depression severity, anxiety severity, and insomnia severity, assessed with a validated questionnaire. The secondary outcome is acceptability, represented by the completion rate.

### 2.2. Search Strategy

The search terms are constructed around four themes (i.e., “mental health”, “cancer”, “mind-body therapies”, and “randomized controlled trial”) using both medical heading (MeSH) terms and text words. The theme “mental health” covers insomnia, depression, and anxiety. The Boolean operator “OR” will be used between search terms within each theme, and the Boolean operator “AND” will be used between search themes. No language restriction will be applied to the search. The literature search will be updated after data collection (including the IPD requests) is completed.

The following databases will be searched until October 2020 (see the full strategy in Appendix A): EMBase, PubMed, Cinahl, Cochrane Library, PsychINFO, IndMED, CSIR-NISCAIR, CNKI, Clinicaltrial.gov, ChiCTR, and CTRI.

Previous reviews will be hand-searched for additional studies and a forward search (i.e., the search of publications that cited the included articles) will be conducted using PubMed. The authors of the included studies will be asked about recent unpublished studies when contacted to request the IPD.

### 2.3. Screening

The studies will be screened by five teams of two reviewers working independently. The results of the screening will be compared between the two reviewers of each team. The first step will be the title/abstract screening. Then the full text of each reference will be downloaded. Finally, the full text will be screened. Disagreements will be resolved through discussion and then through the decision of a third reviewer if consensus cannot be reached. In the case of significant discrepancies, the screening criteria will be revised, and the screening will be conducted again from the beginning.

### 2.4. Data Extraction

The data from the included studies will be extracted by four teams of two reviewers working independently. We will use a standardized data collection spreadsheet for data extraction. We will resolve any disagreements on data extraction through discussion or by consulting a third reviewer, if necessary. The data collection form includes the following items:

- List of authors and contact details
- Year of publication
- Treatment arms with sample size (intention-to-treat)
- Selection of participants according to insomnia, anxiety, or depression diagnosis and diagnostic tool
- Participant characteristics (gender, age, type and stage of cancer, type and stage of anticancer treatment)
- Intervention details (intervention name, treatment frequency and duration, session duration, mode of delivery, presence of home practice) for both experimental and control groups
- Outcome measures
- Outcome at baseline, during treatment, and at post-treatment

### 2.5. Data Request

We will contact the corresponding author of the included studies via email for clarifications. If the corresponding author is not available, we will contact the other authors of the study. If there is no outcome from the email approach, we will contact the authors via phone and social media platforms. We will ask the data provider to remove any identifiable data, such as names, addresses, and/or phone numbers, from the dataset before providing the data. The requested data include the following items:

- Participant covariates (gender, age, type and stage of cancer, type and stage of anti-cancer treatment, presence and type of psychotropic treatment)
- All relevant outcomes for any time point
- Adherence to treatment (number of sessions completed)
- Completion status, with the withdrawal time point and reason in the case of non-completion

For each trial where IPD were supplied, we will reproduce results from trial findings, where possible, and cross-check the data against any published report of the trial. Any inconsistencies will be discussed with the corresponding data providers.

### 2.6. Data Management and Preparation

The data will be stored in a password-protected excel file that is only accessible by the individual responsible for the study (YB) and the statistician (PF). The data will be standardized and collated by YB.

### 2.7. Risk of Bias Assessment

The risk of bias will be assessed by four teams of two reviewers working independently with version two of the Cochrane Collaboration’s Risk of Bias assessment tool [80]. The risk of bias, in terms of the randomization process, deviations from intended interventions, missing outcome data, measurement of the outcomes, selection of the reported results, and overall bias, is judged using the categories of “low”, “some concerns”, or “high”. The risk of bias will be assessed for the primary outcome of the study. In case the information from the report(s) is not sufficient to reach a judgment about the risk of bias, the author of the study will be contacted for clarifications. Disagreements will be resolved through discussion and consulting a third team member reviewer.

### 2.8. Publication Bias Assessment

We will assess potential publication bias with a funnel plot for the whole NMA [81]. The Egger test [82] and the Begg test [83] will also be used to evaluate publication bias.

### 2.9. Heterogeneity and Consistency Assessment

The heterogeneity of the included studies will be presented with the I2 statistic, with values of 50% or more considered to be indicators of a substantial level of heterogeneity [59]. In the case of a high level of heterogeneity, the reason for the heterogeneity (e.g., participant type, intervention type, or duration) will be investigated. Sensitivity analyses will be conducted to determine the impact of these factors on the results of the studies.

In order to comply with the transitivity assumption, only trials in which the interventions could be “jointly randomizable” [84] will be included in the NMA. Inconsistency between direct and indirect sources of evidence will be statistically assessed globally (through the comparison of the fit and parsimony of the consistency and inconsistency models) and locally (through the calculation of the differences between direct and indirect estimates in all closed loops in the network).

### 2.10. Pairwise and Network Meta-Analyses

Traditional meta-analyses will be conducted in cases where several studies have used the same control intervention and the same outcome. Where all studies used the same measurement tool, the results will be combined as mean differences with a 95% CI (for continuous data) and odds ratios with a 95% CI (for dichotomous data). Where studies used different measurement tools, the results will be combined as standardized mean differences (SMDs) with a 95% CI (for continuous data) and odds ratios with a 95% CI (for dichotomous data). A random-effects model will be used for the analysis.

In order to rank the effectiveness of each MBT, an NMA will be conducted. This NMA will include direct and indirect comparisons between different MBTs. The NMA will be adjusted for the presence of multi-arm trials. A network plot will be used to describe the geometry of the treatment network. SMDs with a 95% CI will be used as summary measures. To rank the treatments for each outcome, the surface under the cumulative ranking (SUCRA) probabilities will be used.

All tests will be 2-tailed, and a *p*-value of less than 0.05 will be considered statistically significant. Statistical analyses will be performed using R and/or Stata statistical packages.

### 2.11. Subgroup and Sensitivity Analyses

We will conduct meta-regressions to assess the influence of the following factors on the primary outcomes:

- Cancer site
- Cancer stage, separating early stages (stages 0 and I) and late stages (stages II, III, and IV)
- Presence or absence of concomitant anti-cancer treatment
- Concomitant usage of psychotropic treatment
- Presence or absence of home practice
- Gender

If there is sufficient data, we will conduct sensitivity analyses with the following studies:

- Only studies in which the overall risk of bias was low
- Studies in which the IPD was available and studies in which the IPD was not available (the aggregated mean from the reports will be compared)
- Studies in which mental disorders were diagnosed according to a recognized diagnostic standard

## 3. Discussion

This is the first systematic review in which data from clinical trials testing MBTs for cancer patients is selected according to cut-off scores in psychological scales. This will allow us to provide conclusions about the usefulness of MBTs for cancer patients living with depression, anxiety, and insomnia. The NMA will show the relative effectiveness and acceptability of different MBTs. We will discuss the potential mechanism of MBTs in the discussion section of the future report. The results of this systematic review may have a significant influence on future clinical guidelines and clinical practice. It will inform clinicians about the effectiveness and acceptability of MBTs, thus helping them select the most appropriate intervention for their patients, as well as the treatment dose necessary, to achieve clinically significant changes.

## Data Availability

Not applicable.

## Appendix A

PubMed search (with the filters “Clinical Trial” and “Randomized Controlled Trial” applied).

#1. “mind-body therapies”[MeSH Terms] OR “mind body therap*”[Title/Abstract] OR “yoga”[MeSH Terms] OR “qigong”[MeSH Terms] OR “tai ji”[MeSH Terms] OR “mindfulness”[MeSH Terms] OR “meditation”[MeSH Terms] OR “yoga”[Title/Abstract] OR “qigong”[Title/Abstract] OR “qi gong”[Title/Abstract] OR “taiji”[Title/Abstract] OR “taichi”[Title/Abstract] OR “tai ji”[Title/Abstract] OR “tai chi”[Title/Abstract] OR “mindfulness”[Title/Abstract] OR “relaxation”[Title/Abstract] OR “meditation”[Title/Abstract].
#2. “neoplasms”[MeSH Terms] OR “cancer survivors”[MeSH Terms] OR “neoplasms”[Title/Abstract] OR “tumor”[Title/Abstract] OR “cancer”[Title/Abstract] OR “leukemia”[Title/Abstract] OR “myeloma”[Title/Abstract] OR “lymphoma”[Title/Abstract].
#3. “sleep initiation and maintenance disorders”[MeSH Terms] OR “insomnia”[Title/Abstract] OR “sleeplessness”[Title/Abstract] OR “sleep disturbances”[Title/Abstract] OR “sleep”[Title/Abstract] OR “depressive disorder”[MeSH Terms] OR “depression”[MeSH Terms] OR “depressive disorder”[MeSH Terms] OR “depressive disorder, major”[MeSH Terms] OR “dysthymic disorder”[MeSH Terms] OR “adjustment disorders”[MeSH Terms] OR “depression”[Title/Abstract] OR “mood”[Title/Abstract] OR “anxiety”[MeSH Terms] OR “anxiety”[Title/Abstract] OR “anxiety disorders”[Title/Abstract] OR “phobic disorders”[MeSH Terms] OR “mental health”[MeSH Terms] OR “mental disorders”[MeSH Terms] OR “psycholog*”[Title/Abstract].
#4. #1 AND #2 AND #3.
